# Iatrogenic CJD due to pituitary-derived growth hormone with genetically determined incubation times of up to 40 years

**DOI:** 10.1093/brain/awv235

**Published:** 2015-08-12

**Authors:** Peter Rudge, Zane Jaunmuktane, Peter Adlard, Nina Bjurstrom, Diana Caine, Jessica Lowe, Penny Norsworthy, Holger Hummerich, Ron Druyeh, Jonathan D. F. Wadsworth, Sebastian Brandner, Harpreet Hyare, Simon Mead, John Collinge

**Affiliations:** 1 National Prion Clinic, National Hospital for Neurology and Neurosurgery (NHNN), University College London (UCL) Hospitals NHS Foundation Trust, Queen Square, London WC1N 3BG, UK; 2 MRC Prion Unit, Department of Neurodegenerative Disease, UCL Institute of Neurology, Queen Square, London WC1N 3BG, UK; 3 Division of Neuropathology, NHNN, UCL Hospitals NHS Foundation Trust, Queen Square, London WC1N 3BG, UK; 4 UCL Institute of Child Health, 30 Guilford Street, London WC1N 1EH, UK; 5 Department of Neuropsychology, NHNN, UCL Hospitals NHS Foundation Trust, Queen Square, London WC1N 3BG, UK

**Keywords:** iatrogenic Creutzfeldt-Jakob disease, growth hormone, prion disease, RRP9, PRNP

## Abstract

Patients with iatrogenic Creutzfeldt-Jakob disease due to administration of cadaver-sourced growth hormone during childhood are still being seen in the UK 30 years after cessation of this treatment. Of the 77 patients who have developed iatrogenic Creutzfeldt-Jakob disease, 56 have been genotyped. There has been a marked change in genotype profile at polymorphic codon 129 of the prion protein gene (*PRNP*) from predominantly valine homozygous to a mixed picture of methionine homozygous and methionine-valine heterozygous over time. The incubation period of iatrogenic Creutzfeldt-Jakob disease is significantly different between all three genotypes. This experience is a striking contrast with that in France and the USA, which may relate to contamination of different growth hormone batches with different strains of human prions. We describe the clinical, imaging, molecular and autopsy features in 22 of 24 patients who have developed iatrogenic Creutzfeldt-Jakob disease in the UK since 2003. Mean age at onset of symptoms was 42.7 years. Gait ataxia and lower limb dysaesthesiae were the most frequent presenting symptoms. All had cerebellar signs, and the majority had myoclonus and lower limb pyramidal signs, with relatively preserved cognitive function, when first seen. There was a progressive decline in neurological and cognitive function leading to death after 5–32 (mean 14) months. Despite incubation periods approaching 40 years, the clinical duration in methionine homozygote patients appeared to be shorter than that seen in heterozygote patients. MRI showed restricted diffusion in the basal ganglia, thalamus, hippocampus, frontal and the paracentral motor cortex and cerebellar vermis. The electroencephalogram was abnormal in 15 patients and cerebrospinal fluid 14-3-3 protein was positive in half the patients. Neuropathological examination was conducted in nine patients. All but one showed synaptic prion deposition with numerous kuru type plaques in the basal ganglia, anterior frontal and parietal cortex, thalamus, basal ganglia and cerebellum. The patient with the shortest clinical duration had an atypical synaptic deposition of abnormal prion protein and no kuru plaques. Taken together, these data provide a remarkable example of the interplay between the strain of the pathogen and host prion protein genotype. Based on extensive modelling of human prion transmission barriers in transgenic mice expressing human prion protein on a mouse prion protein null background, the temporal distribution of codon 129 genotypes within the cohort of patients with iatrogenic Creutzfeldt-Jakob disease in the UK suggests that there was a point source of infecting prion contamination of growth hormone derived from a patient with Creutzfeldt-Jakob disease expressing prion protein valine 129.

## Abstract (precis)

Cases of iatrogenic CJD still occur in the UK 30 years after administration of human pituitary-derived growth hormone ceased. Rudge *et al.* report a change over time in genotype profile at polymorphic codon 129 of the human prion protein gene in UK patients, distinct from that seen in other countries.

## Introduction

Prion diseases are neurodegenerative conditions of humans and animals caused by the templated misfolding and aggregation of cellular prion protein (Collinge, 2001). It is becoming increasingly clear that the more common degenerative brain diseases, such as Alzheimer’s and Parkinson’s disease, involve the accumulation of aggregates of misfolded host proteins by a process of seeded protein polymerization (Jucker and Walker, 2013). In this way, prion diseases can be considered a paradigm for the other protein misfolding diseases and prion-like mechanisms are now one of the most rapidly developing research areas in neurodegeneration research internationally. The remarkable and apparently unique phenomenon of zoonotic and horizontal transmission of prions, affords special opportunities to understand the host determinants of clinical phenotype, infection and susceptibility. Genetic variation in the prion protein gene (*PRNP*), specifically a common polymorphism at codon 129 encoding either methionine or valine, is a key risk factor and determinant of susceptibility, clinical phenotype and incubation time (Collinge *et al.*, 1991, 2006; Palmer *et al.*, 1991; Pocchiari *et al.*, 2004; Mead *et al.*, 2006, 2009*a*).

Treatment of persons of short stature with cadaver-sourced growth hormone was first given in 1958 and 1 year later in the UK as a clinical trial (Raben, 1958). In 1985, four cases of Creutzfeldt-Jakob disease (CJD) were reported in patients who had received cadaver-sourced growth hormone in the UK (Powell Jackson *et al.*, 1985). Further cases were reported from the USA, Europe and Australia and treatment with human growth hormone was stopped in the UK in May 1985 by which time recombinant growth hormone was becoming available. Of 1849 persons who received growth hormone in the UK between 1959 and 1985, 38 were known to have developed CJD by 2000 and the estimated risk of developing iatrogenic CJD was at that time 4.5%, this risk being greatest in those patients who received treatment at ages 8–10 years with a peak incubation period of 20 years (Swerdlow *et al.*, 2003). One preparation (Hartree modified Wilhelmi preparation) was common to all patients who developed iatrogenic CJD.

The Medical Research Council (MRC) Prion Unit/National Prion Clinic enrolled two patients with iatrogenic growth hormone related CJD into a clinical trial of quinacrine (PRION-1) (Collinge *et al.*, 2009) between 2002 and 2006 and four others were seen by the Unit over that period. Subsequently the National Prion Monitoring Cohort study (Thompson *et al.*, 2013) was established and between October 2008 and present day (2015) has reviewed nearly all cases of prion disease in the UK including cases of iatrogenic CJD. All cases of iatrogenic CJD were followed up with frequent neurological assessment until death and autopsy confirmation of the diagnosis was made in the majority. This paper reports the clinical and neuropsychological findings, prion protein gene analysis, MRI, EEG, CSF and pathological findings in these cases seen between 2003 and 2014. In light of surprising genetic findings in recent cases we combined our data with those available on older cases for a complete study of the UK experience of cadaveric pituitary growth hormone-related CJD.

## Materials and methods

### Study design

A national referral system for prion diseases was set up in the UK in 2004. UK neurologists were asked by the Chief Medical Officer to refer all patients with suspected prion disease jointly to the National CJD Research and Surveillance Unit (Edinburgh, UK) and to the NHS National Prion Clinic (London, UK). This enables epidemiological surveillance, provision of specialist clinical care and also participation in clinical research and the National Prion Monitoring Cohort study; recruitment of patients to this study began in 2008. All but 2 of 18 patients with CJD in this study who had received cadaver-derived growth hormone were recruited into the Cohort study. In addition, similar patients seen by clinicians in the MRC Prion Unit/NPC as part of the PRION-1 trial of quinacrine (Collinge *et al.*, 2009), between 2001 and 2007, were included in the current study.

After the initial visit, repeated follow-up visits were made to assess progress of the disorder at ∼6-week intervals until death. Progression was assessed by the MRC Prion Disease Rating Scale (MRC Scale), systematic neurological and neuropsychological assessments, and a range of other rating scales (Thompson *et al.*, 2013). Ethical approval for these studies was obtained from the Local Research Ethics Committee of UCL Institute of Neurology/National Hospital for Neurology and Neurosurgery.

### Magnetic resonance imaging

MRI studies performed at either 1.5 or 3 T were reviewed in 20 of the patients. In six of the MRI studies, diffusion weighted images (DWI) were not available and cortical involvement could not be assessed in a comparable way. The images were reviewed by a consultant neuro-radiologist and a consultant neurologist, both with 9 years’ experience in prion disease imaging, and agreement was achieved via consensus review. Signal abnormality was assessed in the caudate, putamen and thalamus on T_2_-weighted, fluid attenuation inversion recovery (FLAIR) and/or DWI sequences. Cortical signal abnormality was assessed on DWI sequences where available in the following areas: frontal, parietal, temporal, occipital, cingulate, insula, hippocampus and cerebellum. Any other relevant findings were also noted.

### Neuropathology

Autopsies were performed either at the local hospital or the National Hospital for Neurology and Neurosurgery. Autopsies were carried out in a post-mortem room designated for high risk autopsies. Informed consent to use the tissue for research was obtained in all cases. The anterior frontal, temporal, parietal and occipital cortex and the cerebellum (at the level of dentate nucleus) were dissected during the post-mortem procedure. The remaining tissue was stored permanently in 10% formalin to allow for additional tissue sampling. In cases where retention of the entire brain was not consented, additional tissue sampling of posterior frontal cortex including motor strip, basal ganglia, thalamus, hippocampus and cerebellar vermis was carried out during post-mortem dissection. Tissue samples were immersed in 10% buffered formalin and prion infectivity was inactivated by immersion into 98% formic acid for 1 h. Tissue samples were processed to paraffin wax and tissue sections were routinely stained with haematoxylin and eosin and immunostained for abnormal prion protein [anti-PrP monoclonal antibody ICSM35 (D-Gen Ltd); 0.1 μg/ml solution] according to published protocols (Wadsworth *et al.*, 2008*c*). The extent of neuropil vacuolation and pattern and intensity of prion protein deposition was assessed semiquantitatively.

### Molecular strain typing of disease-related PrP

Frozen brain tissue was available from seven patients for disease-related prion protein (PrP^Sc^) molecular strain typing (Collinge *et al.*, 1996; Hill *et al.*, 2003). Biochemical studies were carried out in a microbiological containment level 3 facility with strict adherence to safety protocols. Ten per cent (w/v) brain homogenates (grey matter; frontal cortex) were prepared in Dulbecco’s phosphate-buffered saline lacking Ca^2+^ or Mg^2+^ ions using tissue grinders (Wadsworth *et al.*, 2008*c*). Proteinase K digestion (50 µg/ml final protease concentration, 1 h, 37°C), electrophoresis [on 16% Tris-glycine gels (Invitrogen)] and immunoblotting using anti-PrP monoclonal antibody 3F4 (Cambridge Bioscience) was performed as described previously (Wadsworth *et al.*, 2001, 2008*c*; Hill *et al.*, 2003). Internal typing controls of defined PrP^Sc^ types were analysed on each gel when assigning PrP^Sc^ type to an unknown sample. Different human PrP^Sc^ isoforms, referred to as molecular strain types, can be identified in the brain of patients with phenotypically distinct forms of CJD and are classified by both the fragment size and ratio of the three principal PrP bands seen after protease digestion. To date we have characterized four major types of human PrP^Sc^ that can be commonly identified in sporadic and acquired human prion diseases (Collinge *et al.*, 1996; Collinge, 2001; Hill *et al.*, 2003; Wadsworth *et al.*, 2003, 2008*a*, *b*), although much greater heterogeneity seems likely (Hill *et al.*, 2003; Wadsworth and Collinge, 2011). Sporadic and iatrogenic CJD (classical CJD) and kuru are associated with PrP^Sc^ types 1–3, whereas type 4 PrP^Sc^ is uniquely associated with variant CJD. An earlier classification of PrP^Sc^ types seen in classical CJD described only two banding patterns (Parchi *et al.*, 1996, 1999) with PrP^Sc^ types 1 and 2 that we describe corresponding with the type 1 pattern of Gambetti and Parchi and colleagues, and our type 3 fragment size corresponding to their type 2 pattern. More recently Parchi *et al.* (2011) have demonstrated that their PrP^Sc^ types 1 and 2 can be further classified by molecular criteria and have integrated these data with clinical-pathological variations that are seen in patients with classical CJD. Currently the number of distinct prion strains that comprise classical CJD remains unknown and a unified internationally accepted classification system of PrP^Sc^ subtypes remains an important goal (Brandner, 2011).

### Genetic analyses

In addition to the detailed analysis of the clinical data in the 22 patients, which is the main focus of this paper, data were retrieved from the UK iatrogenic CJD database, principally to determine the genotypes in all cases of the polymorphism at codon 129 in the *PRNP* gene. The database contains details of growth hormone preparations received by the patients and duration of disease assessed by the local clinician. Polymorphism at codon 129 was determined in 50 of 77 patients by the MRC Prion Unit or obtained from a previous publication (Swerdlow *et al.*, 2003); another six were assessed by other laboratories. Twenty-one patients did not have gene sequencing or screening for *PRNP* codon 129 polymorphism.

Whole exome sequencing was done using the Agilent SureSelect Human All Exon v2 target enrichment kit. Sequencing was performed on an Illumina HiSeq2000 and achieved an average 30-fold depth-of-coverage of target sequence.

## Results

Details of the individual patient data are shown in Supplementary Table 1. The following is a summary of these data.

### Patients

**Figure 1:**
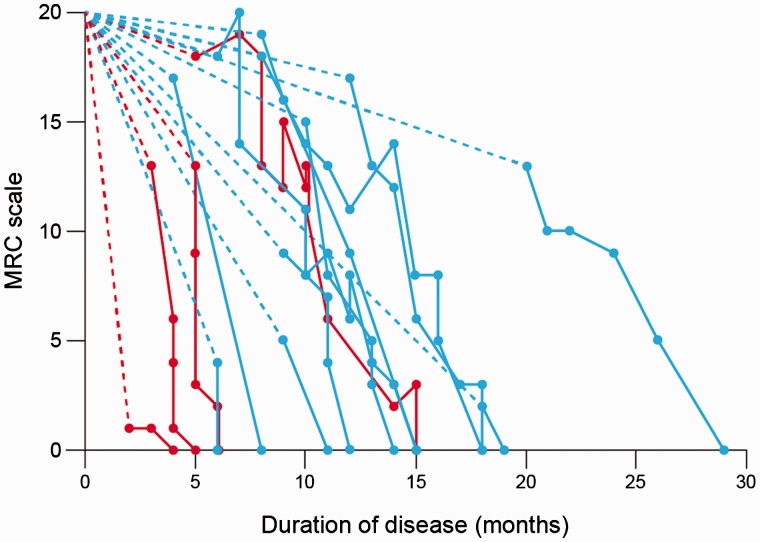
**Clinical progression assessed on the MRC Prion Disease Rating Scale.** Broken line connects time of first symptom to the first assessment on the MRC scale assuming patient would have scored 20 before disease onset. Continuous lines join consecutive assessments indicated by dots. Last assessment is the point at which the patient first scored 0 on the rating scale; death occurred shortly after this in all cases. Horizontal axis = time in months from onset of disease; vertical axis = MRC Prion Rating Scale score; red lines = codon 129 MM patients; blue = codon 129 MV patients.

Twenty-two patients (five female) aged 27–51 (mean 42.8) years were studied sequentially from 2003. All have died. Average follow-up period until death was 7.3 months and the maximum follow-up period was 24.9 months. In a subset of patients, progression was assessed by serial measurements of the MRC Prion Disease Rating Scale (MRC Scale) (Fig. 1). There were between two and 15 follow-up assessments for each patient (average of 6.7 per person); three were assessed twice, one three times, one four times, two five times, one six times and three seven times, one nine times, two 10 times and one 15 times. The mean and median duration of illness was 16.0 (4–32) months and 14.0 months, respectively.

The precise dates of starting and stopping treatment with any type of growth hormone were known for all but one patient; details of treatment with one product (Hartree modified Wilhelmi preparation), which is most implicated as the causative agent of iatrogenic CJD in the UK, were incomplete in two patients. The range of minimum possible incubation times, calculated as the time from last injection of any type of growth hormone to onset of symptoms was 18.3–33.6 (mean 25.9) years and the range of maximum incubation periods calculated from time of first injection to onset of symptoms was 23.2–43.3 (mean 32.8) years, assuming the infection occurred at the first or last exposure. The range of incubation times from midpoint of treatment, ignoring missed injections, with any type of growth hormone to onset of symptoms was 20.6–37.6 (mean 29.3) years. Similar calculations were done for the Hartree modified Wilhelmi preparation product, the only preparation received by every patient, and the most probable source of infection. The range of minimum incubation periods was 22.1–32.8 (mean 27.9) years, maximum incubation period was 22.6–39.8 (mean 31.3) years and the range of incubation periods from the midpoint of injections was 22.5–35.8 (mean 29.9) years. The average and median age at treatment with growth hormone was 9.3 (2–16) years and 9.0 years. The cumulative dose of growth hormone given to each patient is not available but the total duration of treatment is known and has been used as a surrogate measure of total exposure (Supplementary Table 1). There was no relation between duration of dosage of any product and incubation period (r = 0.102, *P* = 0.53 for Hartree modified Wilhelmi preparation; r = 0.08, *P* = 0.61 for all preparations).

### Symptoms

Initial symptoms are summarized in Table 1. Ataxia of gait was the first symptom in 11 of 22 patients and nearly all developed this symptom within the first 2 months. Other initial symptoms were tremor, leg pain, daytime somnolence, dizziness, headaches, myoclonus and cognitive impairment. There were two patients who described unsteadiness induced by a moving stimulus such as waves; one could not stand in the sea due to poor balance and another became unsteady when getting out of shallow water.

**Table 1:** Summary of initial symptoms and neurological signs when first seen

	Initial symptoms		Clinical signs						
Case	First	Second	Gait ataxia	Limb ataxia	Pyramidal	Leg weakness	Myoclonus	Dysarthria	Sensory signs
1	Tremor hands	Unsteady	+	+	+	+	+	+	-
2	Tremor hands	Unsteady	+	+	-	-	-	-	-
3	Tremor/unsteady	Memory decline	+	+	+	+	+	-	+
4	Unsteady	Myoclonus	+	+	+	+	+	+	-
5	Unsteady	Memory decline	+	+	+	+	+	+	-
6	Unsteady	Tremor hands	+	+	-	-	+	-	+
7	Myoclonus	Tremor hands	+	+	+	+	+	+	+
8	Tremor hands	Unsteady	+	+	+	-	+	-	-
9	Unsteady	Somnolence	+	+	+	-	+	+	-
10	Somnolence	Limb pain	+	+	+	-	+	-	+
11	Unsteady	Limb pain	+	+	+	+	+	-	+
12	Unsteady	Myoclonus	+	+	+	-	+	-	+
13	Limb pain	Behaviour/unsteady	+	+	+	+	+	+	+
14	Limb pain	Behaviour/unsteady	+	+	+	+	+	+	+
15	Unsteady	Myoclonus	+	+	+	+	+	+	-
16	Behaviour/memory	Unsteady	+	+	+	-	+	-	-
17	Unsteady	Behaviour	+	+	+	+	-	+	-
18	Dizzy	Unsteady	+	+	+	-	+	+	+
19	Tremor	Unsteady	+	+	-	-	+	+	-
20	Unsteady	Memory decline	+	+	+	+	+	-	+
21	Tremor/unsteady	Limb pain	+	+	+	-	+	+	+
22	Limb pain	Behaviour/unsteady	+	+	+	+	-	-	+

During the evolution of the disease all patients ultimately had unsteadiness of gait and other cerebellar symptoms such as limb dysmetria (lower limb more so than upper limb). Thirteen patients described pain in the lower limbs, including hypersensitivity to light touch in the legs and a variety of dysaesthesiae such as an ‘itchy sensation’, numbness, patchy tingling and burning in the lower limbs. Two had hip pain, three had knee pain, one had both and one patient had calf pain. Ten patients had a sleep disturbance, including daytime somnolence, insomnia and early waking. Two of these patients had excessive sleepiness during the day, falling asleep in public places. There was no pattern of these symptoms occurring at any particular stage throughout the course of the illness.

### Neurological signs

The neurological findings when first seen in the National Prion Clinic are summarized in Table 1. All patients had ataxia of gait, a lesser ataxia of the limbs and most had myoclonus. Half had a cerebellar dysarthria. Seventeen had evidence of pyramidal lesions with either brisk reflexes or extensor plantar responses. Eleven had reduced power in lower limbs.

Relative to the marked ataxia, cognition was initially less affected in most patients. Nineteen patients had a Mini-Mental State Examination assessed at the first visit. Fifteen of these patients scored ≥18/30 (mean 23.7 range 18–29/30), losing points on orientation and recall. One patient could not be tested adequately as she had a history of stable long term cognitive problems. Of the other six patients three were too impaired to test and three scored 8, 7 and 4/30, respectively. There was no correlation between when cognitive function was first assessed and the duration of symptoms. To delineate further the earliest cognitive deficits a short cognitive examination, specifically designed for patients with prion disease, was done in the nine patients well enough to cooperate. Executive function assessed by letter fluency (1 min) was mildly impaired in all when first tested 3–19 (mean 8.4) months after the first symptoms. Verbal or visual recognition memory was mildly impaired in the three patients seen more than 1 year after onset of symptoms. All had preserved visual perception. Four patients had more extensive psychometric testing confirming mild memory impairment in all and mildly impaired executive function in three. Subsequently there was progressive decline in cognitive function; increasing dysarthria limited assessment later in the course of the disease.

Over a period of months the ataxia increased resulting in loss of ambulation and inability to groom, dress and feed without assistance. Leg weakness increased and contributed to the loss of ambulation. The dysarthria progressively increased and speech became unintelligible. Speech output then gradually declined making cognitive assessment difficult. Myoclonus usually persisted but the pain in the limbs lessened in most patients. Incontinence of urine and faeces developed in all. Ultimately the patient entered an akinetic mute state.

### Magnetic resonance imaging

**Figure 2:**
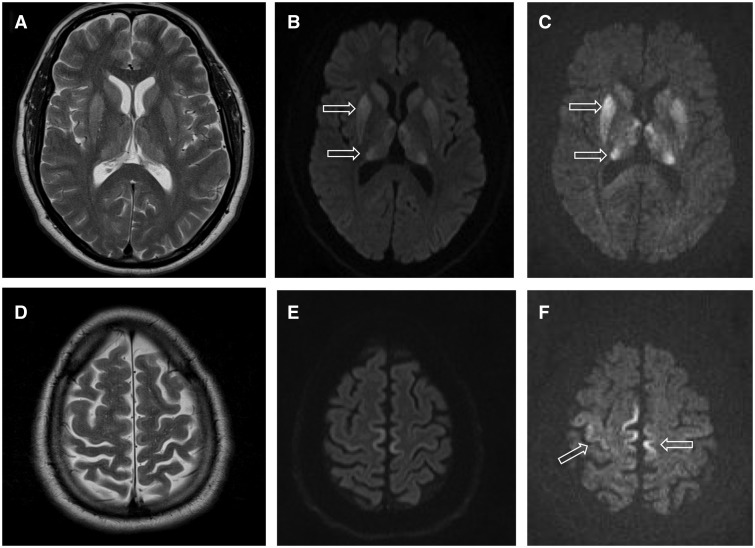
**MRI findings in a 46-year-old male patient with iatrogenic CJD due to growth hormone**. Basal ganglia and thalamic hyperintensity is seen on T_2_-weighted images (**A**), DWI at b = 1000s/mm^2^ (**B**) and (**C**) DWI at b = 3000s/mm^2^. Cortical hyperintensity in the paracentral lobule bilaterally and right precentral gyrus is not visualized on T_2_-weighted images (**D**) but is seen on DWI b = 1000s/mm^2^ (**E**) and (**F**) is more conspicuous at DWI at b = 3000s/mm^2^ (Patient 12).

**Figure 3:**
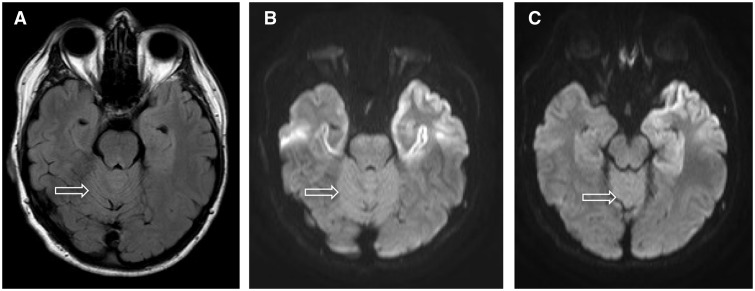
**MRI findings in a 47-year-old male patient with iatrogenic CJD due to growth hormone.** Hyperintensity in the superior cerebellar vermis is seen on axial FLAIR images (**A**), and is more conspicuous at DWI at b = 1000s/mm^2^ at the same level (**B**). (**C**) A more superior axial image at DWI at b = 1000s/mm^2^ shows the increased vermian signal abnormality compared to the surrounding occipital cortex (Patient 8).

MRI brain scans were available in 20 patients (five female) mean age 42.7 years (range 27–51 years). Eighteen of the patients scanned showed caudate, putamen and thalamic signal abnormality (Fig. 2 and Table 2). The thalamic signal abnormality was seen in all the patients where there was basal ganglia signal abnormality and was mostly diffuse with clear involvement of all the thalamic nuclei. Of the cortical areas, the cingulate gyrus most commonly showed signal abnormality (15 of 17 patients who underwent DWI). The frontal cortex was next most commonly involved (14 of 17 patients), with focal involvement of the medial aspect of the precentral gyrus and paracentral lobule bilaterally seen in 10 of 16 (Fig. 2) with relative sparing of the postcentral gyrus. In five of these patients, the abnormality was confined to the precentral gyrus. The next most commonly affected cortical area was the superior cerebellar vermis, seen in 7 of 16 patients (Fig. 3). The parietal, temporal and insula cortices were rarely involved and the occipital cortex was not involved in any of the cases (Table 2). Involvement of the hippocampus, particularly the tail, was seen in nine patients. Additional MRI findings included one patient with septo-optic dysplasia and partial agenesis of the corpus callosum.

**Table 2:** Summary of MRI findings in each patient

Case	MRI	Caudate	Putamen	Globus Pallidus	Thalamus	Frontal	Paracentral Lobule	Parietal	Temporal	Occipital	Cingulate	Insula	Hippo campus	Cerebellum
1	Y limited	+	+	-	+	n/a	n/a	n/a	n/a	n/a	n/a	n/a	n/a	n/a
2	Y limited	-	-	-	-	+	-	-	-	-	+	-	+tail	+
3	Y	+	+	-	+	+	+	-	-	-	+	-	+	-
4	Y limited	+	+	-	+	n/a	n/a	n/a	n/a	n/a	n/a	n/a	n/a	n/a
5	N													
6	Y limited	+	+	-	+	n/a	n/a	n/a	n/a	n/a	n/a	n/a	n/a	n/a
7	Y	+	-	-	+	+	+	-	-	-	+	+	-	+
8	Y	+	+	-	+	+	-	-	+	-	+	+	+tail	+
9	Y	+	+	-	+	+	+	-	-	-	-	-	+tail	+/-
10	Y limited	+	+	-	+	+	n/a	+	-	-	+	-	-	+
11	Y	+	+	-	+	+	+	-	-	-	+	-	-	+
12	Y	+	+	-	+	+	+	+	-	-	+	-	-	+/-
13	Y	+	+	-	+	-	-	-	-	-	+	-	+	-
14	Y	+	+	-	+	-	-	-	-	-	+	-	-	+
15	Y limited	+	+	-	+	+	+	+	+	-	+	-	+tail	-
16	Y	+/-	+/-	-	+/-	-	-	+	-	-	-	-	-	+/-
17	N													
18	Y	+	+	-	+	+	-	+	+	-	+	+	+head	+
19	Y	+	+	-	+	+	+	-	-	-	+	-	-	+
20	Y	+	+	-	+	+	+	-	+	-	+	-	+tail	+
21	Y	+	+	-	+	+	+	-	-	-	+	-	+/-	+/-
22	Y	+	+	-	+	+	+	+	-	-	+	-	+	-

Y = MRI obtained; N = MRI not obtained; Y limited = limited or no DWI sequences available;+ = feature present; - = feature absent; +/- = equivocal abnormality.

n/a = not available.

### Prion protein gene sequencing

All patients were analysed for the entire *PRNP* open reading frame by Sanger sequencing. None had a mutation. At polymorphic codon 129 there were 17 heterozygotes (MV), four methionine homozygotes (MM) and one valine homozygote (VV).

Although there were insufficient data to compare statistically survival times by 129 polymorphism, MM patients had a mean duration of 7.8 months, the VV patient 17 months and those with the MV polymorphism a mean of 18.6 months (10–32 months). In addition, the duration of disease from first symptom was significantly longer in the heterozygotes (*P* < 0.02 two-tailed *t*-test). Although there were only four patients who were MM three of these had the most rapid progression (*P* = 0.04, Mann Whitney U-test).

The average incubation time from the midpoint of growth hormone administration was 28.6 years for MV and 31.8 years for MM (four patients). The patient who carried the VV polymorphism developed symptoms after 20.6 years.

**Figure 4:**
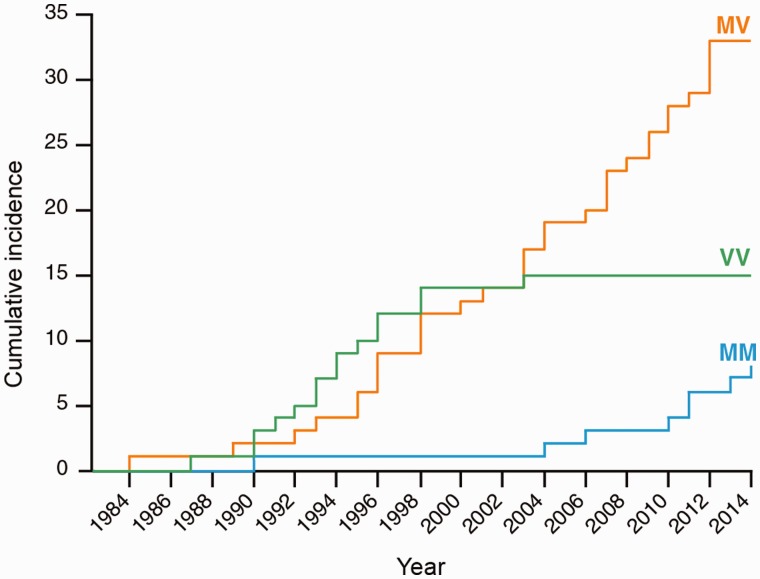
**Cumulative incidence of patients stratified by *PRNP* codon 129 polymorphism.** There is a highly significant difference in the distribution of the three polymorphisms at codon 129 over time and incubation period (ANOVA, *P* < 10^−7^), with each pairwise comparison of genotypes being statistically significant in *post hoc* analyses (*P* < 0.001) as indicated in the text.

Combining the data with those already published (cases up to 2000) there were 33 heterozygotes (MV), and 23 homozygotes, 15 of whom were VV and eight MM. There was a highly significant difference in the distribution of the three polymorphisms at codon 129 over time and incubation period, with each pairwise comparison of genotypes being statistically significant in *post hoc* analyses [MM mean 30.8 years (95% confidence interval 26.9–32.6), MV mean 23.4 years (9.0–36.7), VV mean 14.3 years (7.7–20.2), ANOVA *P* < 10^−7^]. Fourteen of 15 valine homozygotes occurred before 1998 and seven of eight methionine homozygotes occurred after 2004 (Fig 4).

### Whole exome sequencing

Twenty-seven iatrogenic CJD cases, including the 22 in this report and one from outside the UK, were exome sequenced and allele frequencies compared with internal and publicly available control frequencies. The top-ranked single variant association was in *RRP9* (ribosomal RNA processing 9 small subunit gene), for which association testing of rs145537768, p.R143W, achieved *P* = 10^−7^, Fisher’s exact test. While this was the strongest statistical association in the exome study it does not surpass standard statistical thresholds for a genome-wide finding (*P* < 5 × 10^−8^) and requires replication study in other national cohorts of iatrogenic CJD to be recognized as a confirmed association. The variant was detected in five growth hormone-related iatrogenic CJD cases (*n* = 27), confirmed in all cases by Sanger sequencing, but was not found in 574 internal non-prion disease controls. This variant changes a conserved amino acid residue, is predicted to be damaging by both *in silico* prediction software PolyPhen-2 and SIFT, and is rare in control populations with a reported allele frequency of 0.0014 in non-Finnish Europeans (122/121370 in the entire series, http://exac.broadinstitute.org/gene/ENSG00000114767).

### Electroencephalography

Twenty-one patients had an EEG, six of which were initially normal. The other 15 had generalized slow activity and one of which had short runs of periodic slow wave complexes.

### Nerve conduction studies

No systematic peripheral nerve conduction studies were undertaken. However, four patients had been investigated by the local clinician prior to referral to the National Prion Clinic. One patient with wasting of the small hand muscles and fasciculation had neurophysiological evidence of denervation leading to an initial diagnosis of motor neuron disease. A second patient had clinical evidence of a length dependent sensorimotor axonal neuropathy, greater in the lower limbs, assessed 1 year before referral, while two other patients also had some evidence of a similar sensory motor neuropathy on limited assessment.

### Cerebrospinal fluid

Sixteen patients had CSF examination. The fluid was acellular in all. Five had a raised protein concentration (>0.41 g/l). 14-3-3 protein was sought in 14 patients and detected in six.

### Neuropathology

**Figure 5:**
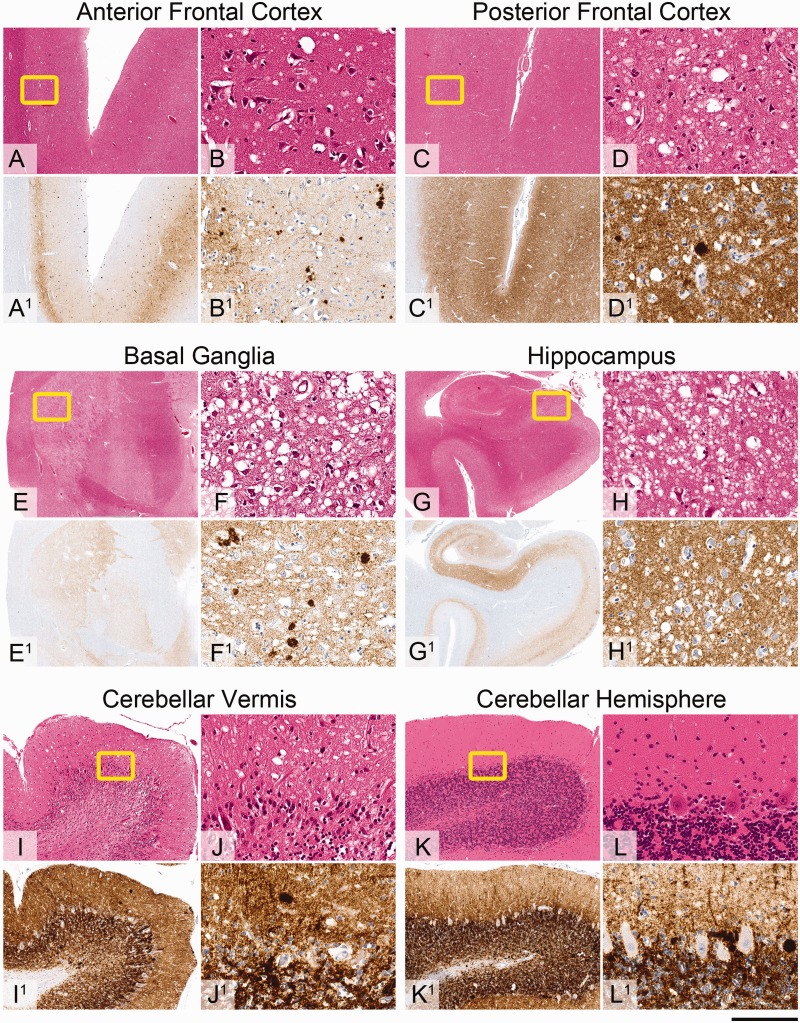
**Neuropathological findings in a representative iatrogenic CJD case.** Sections (**A–L**) stained with haematoxylin and eosin. Sections (**A^1^**–**L^1^**) immunostained for abnormal prion protein deposition with anti-PrP monoclonal antibody ICSM35. Throughout the grey matter there is variably prominent microvacuolar degeneration and abnormal prion protein deposition in a diffuse synaptic manner and as micro-plaques. In the anterior frontal cortex (**A** and **B**, haematoxylin and eosin) there is mild patchy microvacuolar degeneration and abnormal prion protein deposits (**A^1^** and **B^1^**, ICSM35) are restricted to the deep cortical layers. In the posterior frontal lobe, including motor cortex (**C** and **D**, haematoxylin and eosin) there is more prominent micro-vacuolation and dense pan-cortical deposition of the abnormal prion protein (**C^1^** and **D^1^**, ICSM35). In the basal ganglia (**E**, haematoxylin and eosin; **E^1^** ICSM35) there is very severe vacuolation and diffuse deposition of abnormal prion protein in the neuropil of the putamen (**F**, haematoxylin and eosin; **F^1^** ICSM35) and caudate nucleus. In the hippocampus (**G**, haematoxylin and eosin) there is particularly prominent micro-vacuolar degeneration in the Sommer’s sector, subiculum and entorhinal cortex (**H**, haematoxylin and eosin) with corresponding deposits of abnormal prion protein (**G^1^** and **H^1^**, ICSM35). In the cerebellum there is very severe cortical atrophy in vermis with prominent microvacuolation in the molecular layer, loss of Purkinje cells, granular cells and accompanying Bergmann gliosis (**I**, haematoxylin and eosin). The cortex in the cerebellar hemisphere is much better preserved with mild degree of microvacuolar degeneration in the molecular layer (**J**, haematoxylin and eosin). In both, cerebellar vermis and hemisphere there are scattered micro-plaques and diffuse, dense synaptic labelling for abnormal prion protein. Scale bars = 4 mm in **A**, **A^1^**, **C**, **C^1^**, **E**, **E^1^**, **I**, **I^1^**, **K** and **K^1^**; 8 mm in **G** and **G^1^**; 200 µm in **B**, **B^1^**, **D**, **D^1^**, **F**, **F^1^**, **H**, **H^1^**, **J**, **J^1^**, **L** and **L^1^**.

Nine autopsies were conducted between 2003 and 2013; the whole brain was available in eight. We report an overview here; a full morphological study will be reported separately. In all cases there was variably prominent microvacuolar degeneration in the hemispheric cortex, deep grey nuclei and cerebellar cortex. Immunostaining for the abnormal PrP revealed synaptic labelling in all grey matter areas examined and in all but one case, there were also microplaques in all grey matter structures (Fig. 5). Variability in the intensity of the immunoreactivity for the abnormal prion protein was evident but detailed comparison between the cases and separately within each case was not feasible as prolonged formalin-fixation in some cases significantly attenuated the immunoreactivity.

### Disease-related prion protein analysis by immunoblotting

**Figure 6:**
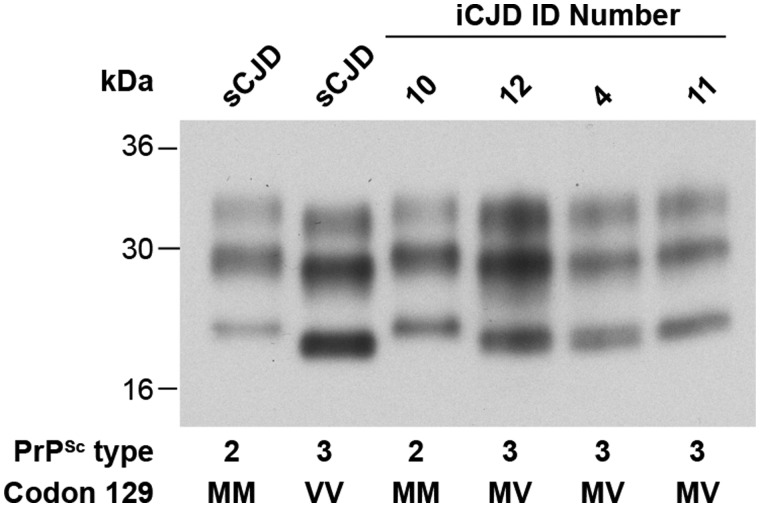
**PrP immunoblot of patient brain.** Proteinase K (PK)-digested 10 % (w/v) brain homogenates from patients with sporadic CJD or iatrogenic CJD were analysed by enhanced chemiluminescence using anti-PrP monoclonal antibody 3F4. The provenance of each brain sample is designated above each lane and the type of PrP^Sc^ (London classification; Hill *et al.*, 2003) detected in each sample and the *PRNP* codon 129 genotype of the patient (M, methionine; V, valine) is designated below. Control samples of human PrP^Sc^ types 2 and 3 from patients with sporadic CJD (lanes 1 and 2) are compared with the PrP^Sc^ molecular strain types seen in Patients 4, 10,11 and 12 with iatrogenic CJD (lanes 3–6).

PrP^Sc^ typing (molecular strain typing) (Collinge *et al.*, 1996; Hill *et al.*, 2003) was performed with frontal cortex samples from seven patients with iatrogenic CJD where frozen tissue was available. A representative immunoblot is shown in Fig. 6. All seven cases showed PrP^Sc^ types that are congruent with those seen in sporadic CJD. Five were 129MV with type 3 PrP^Sc^ and two were 129MM with type 2 PrP^Sc^ using the London classification (Hill *et al.*, 2003).

## Discussion

This study, covering the period from 2000–14, shows that iatrogenic CJD due to cadaver-sourced pituitary growth hormone, a treatment that was discontinued in 1985 in the UK, continues to occur in the UK at a frequency of 0–6 cases per annum. Incubation periods are now extraordinarily long, with estimates ranging from 18–40 years, the uncertainty based on clinical onsets and lengths of treatment with potentially infected batches. In this paper we have reviewed the clinical features, progression, imaging abnormalities, prion protein genotype, PrP^Sc^ type by western blot and preliminary neuropathology data.

### Clinical features and imaging correlations

Typically patients with growth hormone induced iatrogenic CJD present with gait ataxia, cerebellar dysarthria and lower limb pain with cognitive function much less affected. Later sleep disturbance, cognitive decline and pyramidal signs with weakness in the lower limbs develops. Ultimately, the patient enters an akinetic mute state typical of all types of CJD with death at 4–32 (mean 16.0) months after the initial presentation. There are several points of special interest in this clinical presentation.

First, gait ataxia, with much less limb ataxia, especially in the upper limbs, is typical of superior cerebellar vermis damage. The MRI and pathological examination of the cerebellum demonstrated extensive damage of the vermis with lesser involvement of the cerebellar hemispheres consistent with these clinical findings. Interestingly, in addition to gait ataxia, two of the patients presented with abnormal visual input imbalance characterized by unsteadiness induced by moving stimuli (waves when paddling and looking at moving water) suggesting abnormal visual vestibular interaction partly dependent on cerebellar pathways.

Second, pain in the lower limbs was of a quality suggesting abnormal spino-thalamic function and MRI confirmed a diffuse abnormality of the thalamus which, although maximal medially, did extend laterally to the ventro-posterior components, part of the pain and thermal pathways. Of note is the similarity of this pain to that experienced by many patients with variant CJD and kuru, which are also acquired prion diseases associated with peripheral infection with prions where thalamic involvement, especially posteriorly, occurs. However, a contribution of the more peripheral components of the spino-thalamic system cannot be excluded.

Third, later in the course of the disease cognitive dysfunction with prominent memory decline emerged. The extensive involvement of many areas of the deep nuclei, thalamus and cortex could explain this, but of note is the prominent involvement of the hippocampus on MRI and pathologically. This, together with the thalamic changes, is an adequate explanation of the amnesia. Thalamic involvement would also explain the sleep disturbance similar to that occurring in fatal familial insomnia (FFI) and frequently seen in sporadic CJD.

Finally, weakness in the lower limbs due to pyramidal involvement correlated with cortical ribboning involving the dorsal and medial motor strip demonstrated on MRI in most patients. Such involvement of the motor cortex is rare in other forms of prion disease. More pronounced involvement of the motor cortex and parietal lobe when compared with the anterior frontal or occipital cortex was also confirmed morphologically with extensive micro-vacuolar change and intense deposition of synaptic abnormal prion protein.

### Neuropathology

A detailed quantitative analysis of the pathology in the nine autopsied cases will be the subject of a separate communication. However, a striking feature was the presence of PrP plaques in all but one case. This latter non-plaque case suggests that the different pathological appearances might be caused by different prion strains. Similarly, iatrogenic CJD due to dural grafts, a condition particularly prevalent in Japan, may show two distinct pathologies, plaque-type and non-plaque-type, which have been linked with differing transmission properties in transgenic mice (Kobayashi *et al.*, 2014). It is possible that future studies using material from the original batches of pituitary-derived growth hormone and transgenic mice could clarify the pathogenesis of the disease in our cases and comparison with the literature on dural graft iatrogenic CJD.

### *PRNP* codon 129 polymorphism determines incubation time

A remarkable feature of iatrogenic CJD cases in the UK is the distribution of the codon 129 polymorphism. An excess of VV homozygotes was reported in early UK cases and then thought to be a marker of susceptibility (Collinge *et al.*, 1991). MV heterozygosity was reported to confer relative resistance to sporadic CJD (Palmer *et al.*, 1991). In the initial study of 27 UK patients 52% were homozygous for valine and only 4% (one case) was homozygous for methionine; the remainder were heterozygotes. This contrasts with the French experience in 77 patients where 48% were homozygous for methionine and 22% homozygous for valine (Brandel *et al.*, 2003). Similar proportions were also found in the few cases reported from the USA (Brown *et al.*, 2012). Furthermore in sporadic CJD, kuru and variant CJD, methionine homozygosity predominates. This is thought to result from the relative resistance afforded by heterozygosity at polymorphic PrP residue 129 and from conformational selection whereby different prion strains are preferentially propagated by prion proteins of different primary sequence (Collinge, 1999; Collinge and Clarke, 2007). For example the bovine spongiform encephalopathy-related prion strain causing variant CJD appears only to be compatible with methionine 129 human PrP (Wadsworth *et al.*, 2004).

One of the new findings of our study of UK patients is that there has been a change in the distribution of the 129 polymorphism in the past 12 years. The VV genotype has now greatly decreased and MM genotype increased while the frequency of heterozygotes has remained relatively constant. There are similarities and differences from the studies of kuru (Cervenakova *et al.*, 1999; Collinge *et al.*, 2006; Mead *et al.*, 2009*b*). In kuru both homozygous genotypes predominated in young cases with presumably a shorter incubation time, but in later and older cases heterozygotes occurred more frequently.

A possible explanation for these superficially contradictory distributions of codon 129 in different outbreaks could be the compatibility of host genotype and strain of the infecting prion, in keeping with the conformational selection model of prion transmission (Collinge, 1999; Collinge and Clarke, 2007). This model suggests that transmission is facilitated, with shorter incubation times, if the host prion protein can readily adopt the preferred conformation associated with the strain of the infecting prion. In the case of the UK, it is likely that one particular preparation (Hartree modified Wilhelmi preparation) was responsible for the outbreak as all patients to date received this preparation.

Swerdlow *et al.* (2003) estimated that ∼400 000 pituitaries were harvested for growth hormone production, but even this may be an underestimate. Attempts were made to exclude patients who had neurological diseases but protocols for harvesting were not strictly monitored (Swerdlow *et al.*, 2003). It is likely that some of these pituitary glands were sourced from cases with CJD; in the 1970s, the time when the majority of pituitary sourced growth hormone was obtained, it is estimated from UK mortality data that 1 in 7000 deaths would have been due to sporadic CJD. Two-thirds of cases of sporadic CJD worldwide are of the 129MM genotype and therefore it is not surprising that most cases of iatrogenic CJD outside the UK were of this genotype. However, in the present series, the pattern was different with the early patients, which were predominantly of the VV genotype. As only ∼24% of cases of sporadic CJD are 129VV the question arises as to why the UK patients differ from the rest of the world in this regard.

One possibility is that the screening of donors was more successful than suggested above and by chance only one or two cases of sporadic CJD were the source of the growth hormone and these had an atypical phenotype that occured more frequently in VV or MV cases. Alternatively many donors were included, however, a single VV or MV case had a particularly high titre of infective material in the pituitary. The profound influence of codon 129 in controlling human prion transmission barriers has been extensively modelled in transgenic mice expressing human PrP on a mouse PrP null background (Wadsworth *et al.*, 2010). These studies have shown that transmission barriers to infection with classical CJD prions are asymmetric, dependent upon the codon 129 genotype of the prion source and the recipient (Wadsworth *et al.*, 2010). Whereas, transgenic mice expressing human PrP 129 valine are highly susceptible to classical CJD prions regardless of the PrP^Sc^ type or codon 129 genotype of the inoculum (Collinge *et al.*, 1995, 1996; Telling *et al.*, 1995; Hill *et al.*, 1997; Korth *et al.*, 2003; Kobayashi *et al.*, 2007; Wadsworth *et al.*, 2008*b*), the absence of a transmission barrier to classical CJD prions is not uniformly observed in mice expressing human PrP 129 methionine in the absence of mouse PrP. Here, mismatch at residue 129 between the inoculum and host can significantly affect transmission. Thus while there appears to be no barrier to transmission of classical CJD prions from codon 129 methionine homozygous patients (Asante *et al.*, 2002; Korth *et al.*, 2003; Beringue *et al.*, 2008; Kong *et al.*, 2008), transmission of classical CJD prions from valine homozygous patients is often associated with more prolonged and variable incubation periods and reduced attack rates (Asante *et al.*, 2002; Korth *et al.*, 2003; Kobayashi *et al.*, 2007, 2015). In the case of classical CJD prions from codon 129 heterozygous patients the transmission efficiency in transgenic mice expressing human PrP 129 methionine varies dependent upon PrP^Sc^ type and whether prions are propagated on human PrP with methionine or valine at residue 129 (Asante *et al.*, 2002; Korth *et al.*, 2003; Bishop *et al.*, 2010; Kobayashi *et al.*, 2015). These data provide an experimental background with which to interpret the temporal distribution of codon 129 genotypes within the cohort of iatrogenic CJD patients in the UK and suggest that the infecting prion contamination of growth hormone was from a VV or MV individual.

Finally, the significance of the polymorphism in *RRP9* found on exome sequencing is unclear. The sample is small and the finding requires replication in an independent cohort. Any association could be related to either the initial condition for which the patients were treated or iatrogenic disease or both.

### Mismatch of incubation period and rapidity of symptomatic progression

A further seemingly paradoxical observation is that those recent clinical cases with particularly long incubation times have had the shortest clinical durations once symptomatic. Rates of clinical decline in our series of iatrogenic CJD have been most in keeping with observations of sporadic CJD with the MM genotype being more rapid than MV (Pocchiari *et al.*, 2004). A plausible explanation for these observations is that the generation of a host prion strain compatible with host genotype occurs in the periphery during the prolonged incubation time; however, following CNS invasion the rapidity of disease progression [which is thought to be determined by the rates of production of toxic PrP species (designated PrP^L^ for lethal); Hill *et al.*, 2000; Collinge and Clarke, 2007; Sandberg *et al.*, 2011, 2014] is more rapid in MM versus MV individuals owing to higher levels of homotypic substrate PrP available for conversion.

## Conclusion

This study is the first to clearly define the clinical, imaging and neuro-pathological characteristics of patients with iatrogenic CJD due to cadaver-sourced growth hormone. It demonstrates that cases continue to occur at a low but steady rate in the UK and that the incubation period can be up to four decades. We have shown that all three common genotypes at *PRNP* are susceptible albeit with markedly different incubation periods, a phenomenon also seen in kuru. Whether similar susceptibility, with differing mean incubation times, will be seen in the UK related to the transmission of bovine spongiform encephalopathy prions remains to be seen. Further, we have demonstrated a dissociation between the incubation period and the rapidity of decline once a person develops symptoms.

## Supplementary Material

Supplementary Table 1

## Abbreviations

CJD: Creutzfeldt-Jakob disease
DWI: diffusion-weighted image
PrP: prion protein
PrP^C ^: normal cellular form of prion protein
PrP^Sc ^: ‘scrapie’ or disease-associated isoform of prion protein
